# Delayed Access to Dementia Services: The Experiences of the African Caribbean Community in the UK

**DOI:** 10.1177/14713012251381009

**Published:** 2025-09-12

**Authors:** Melissa G. Brown, Stephanie L. Howarth, Gerard A. Riley

**Affiliations:** 1 School of Psychology, 1724 University of Birmingham, UK

**Keywords:** dementia, services, African Caribbean, barriers, culture, access, help seeking, ethnicity

## Abstract

African Caribbean people in the UK have a higher incidence of dementia compared to White people. Despite this, they are less likely to seek help from services. Previous research has investigated barriers to help-seeking for dementia in ethnic minorities, but this research has typically involved samples drawn from a range of ethnic groups. Given their increased risk, it would be useful to focus specifically on the African Caribbean community. Fourteen African Caribbean participants, with experience of supporting a family member with memory loss, participated in an interview exploring their perceptions of barriers to help-seeking. Reflexive Thematic Analysis was used to guide the collection and analysis of interview data. Historical experiences of the African Caribbean community have made them self-reliant and stoical in the face of adversity, and unwilling to seek help, particularly from a society that rejected them. Memory loss is likely to be attributed to old age or harmless individual traits, and the concept of dementia is not well rooted in the culture. Shame and stigma attached to mental difficulties in the culture also make people unwilling to seek help and motivated to mask their difficulties. There are cultural and familial expectations that care should be provided by daughters within the family, not by outsiders. Participants were reluctant to make use of services due to concerns that, because of racism, the family member will receive a poorer quality service, a culturally insensitive service, or be mistreated. There is a need to raise awareness and understanding of dementia in this community, and to tackle the stigma associated with it. Ongoing racism and cultural insensitivity within services also need to be addressed. Because of the community’s mistrust of government and mainstream institutions, African Caribbean community organisations should be funded to lead efforts to deal with these issues.

## Introduction

Dementia affects those from Black communities in the UK at a higher rate compared to White and Asian communities (Adelman et al., 2009; Mukadam et al., 2023; Pham et al., 2018). This has been attributed to higher rates of dementia-related risk factors such as hypertension, diabetes, obesity, and socio-economic deprivation (Adelman et al., 2009; Livingston et al., 2020).

Despite this higher rate, those from Black communities and other ethnic minorities are more likely to experience a missed diagnosis, to receive their diagnosis at a later stage than those from the White community, and less likely to make use of dementia services (Dodd et al., 2022; Ogliari et al., 2020). In part, this may be due to service-related biases that result in these processes being initiated and conducted less efficiently or effectively for people from these communities – e.g., language-related issues such as lack of interpreters and the unavailability of cognitive assessments in languages other than English (Brown et al., 2021). In part, it may be due to delays in seeking help from services or in accessing services that are available (Johl et al., 2016; Mukadam et al. 2011b).

This is an important issue. Early diagnosis of dementia has many benefits (Rasmussen & Langerman, 2019). For example, the disease-slowing medications that have recently been approved for use in Alzheimer’s disease are only effective for the earlier stages of the disease (Wahlberg et al., 2024). People from Black communities are also disadvantaged if, after diagnosis, they make less use of support services available for those living with dementia.

Several studies have examined the reasons for the delay amongst ethnic minorities in seeking help initially and in making use of dementia services once diagnosed. These include a lack of understanding of dementia and its differentiation from the forgetfulness associated with normal ageing; a lack of awareness of what services can offer and an associated belief that there would be no advantage to seeking help; cultural expectations about providing care within the family; cultural stigma associated with mental dysfunction and impairment; and expectations of cultural insensitivity, racism and discrimination within health and social care services (Baghirathan et al., 2020; Johl et al., 2016; Mukadam et al., 2011a, 2011b, 2015; Victor et al., 2024).

Most of these studies of delayed help-seeking have used samples containing a range of different ethnic minorities. Although this is useful in terms of painting a broader picture, studies focused on specific community groups are also needed because different groups are likely to have different motivations for delay. This idea is supported by evidence about other cases of delayed access to health services by ethnic minorities. For example, in UK studies of hesitancy about getting a COVID vaccine, ethnic groups differed in the extent to which hesitancy occurred and in the reasons for hesitancy (Hussain et al., 2022).

There appears to be only one previous study that has focused exclusively on the UK Black community. Berwald et al. (2016) ran focus groups for participants from Black and African Caribbean communities across the UK. Participants were not diagnosed with dementia and were not required to have any prior knowledge or experience of dementia or dementia services. They were presented with a vignette of an older person from their community presenting with memory difficulties, and asked to discuss how they would help a member of their family in a similar situation, where they would seek help from, and what would encourage or discourage their help-seeking. Their findings offer more nuanced and detailed examples of the reasons outlined in the broader mixed-ethnicity literature. For example, related to the broader finding that a lack of understanding about dementia underlies delayed help-seeking, some participants in this study viewed dementia as an illness of White people. Some considered it would be a problem to discuss private and stigmatising problems with strangers and considered that help and support should come from within the family. Some felt there was little to be gained from seeking advice from services, and some were concerned about harm from medication or forced detention.

Further exploration of the reasons for delayed help-seeking in Black communities is warranted. Building on the study by Berwald et al. (2016), there would be an advantage in exploring the experiences of those who have actual experience of dealing with a family member with memory problems. Furthermore, even within Black communities, there may be differences between those with a Caribbean heritage and those of direct African origin. Previous studies have suggested that migration influences help-seeking for dementia (Jutlla, 2015; Victor et al., 2024) and those with a Caribbean heritage have historical experiences associated with migration that are specific to this community. Those currently at risk of developing dementia within this community because of their age belong to the *Windrush Generation*. These are people who migrated from the Caribbean to the UK between 1948 and 1971, invited by the government to help rebuild Britain after the Second World War. Despite being invited and despite their contribution to society, the community experienced severe racism and discrimination. Although there were improvements over time (e.g., the Race Relations Act in 1965 and 1968 outlawed discrimination based on ethnicity), the community have undergone a more recent experience of rejection and exclusion. In 2012, the UK government introduced a ‘hostile environment’ policy requiring people to provide proof of their right to reside in the UK (UK Government, 2024). Those unable to do so faced deportation and exclusion from access to employment, health and social care, housing etc. Many of the Windrush Generation were unable to provide the necessary evidence, in large part because the UK government had destroyed the original documentation. Some were wrongly deported and many experienced hardships because of being unable to access services. The errors and mistreatment were eventually acknowledged by the UK government and a compensation scheme established. However, there continues to be a sense of grievance relating to the inadequacy of the compensation, and the slowness and bureaucracy of the process (UK Government, 2024).

The aim of the present study, therefore, was to explore the help-seeking experiences of those of Caribbean heritage with direct involvement in supporting someone with dementia. A particular focus of the study was an exploration of how being part of the Windrush Generation may impact help-seeking for dementia.

## Method

The study was approved by a research ethics committee of the UK’s Research Ethics Service (IRAS ID: 319096). Participants provided fully informed written consent.

### Recruitment

Participants were recruited from the West Midlands area of the UK. This is an area of significant cultural diversity with several large cities and towns. The largest city, Birmingham, has a higher percentage of people identifying as African Caribbean than any other local authority in the UK (UK Government, 2019). The study was advertised on posters displayed in places identified as being used by the African Caribbean community, including community centres, charity premises, churches, and business premises. Recruitment material was also displayed in the premises of local NHS Memory Assessment Services and other dementia-related services, and clinicians were asked to draw attention of potential participants to the material.

Participants were required to identify as being African Caribbean or of African Caribbean descent, at least 16 years of age, and capable of taking a meaningful part in an interview conducted in English. They were also required to be in regular contact with a family member who identified as African Caribbean or African Caribbean descent, and who was experiencing significant memory loss.

The aim was to recruit a sample of between 6 and 15 participants, which is the range recommended for smaller-scale research projects involving Reflexive Thematic Analysis (Braun & Clarke, 2022).

### Participants

Fourteen people took part, with three taking part in one interview because they were all related to the same person with memory loss. Demographic and other information about each participant in shown in Table 1. Twelve of the participants identified as being of African Caribbean, and two as mixed African Caribbean and White.

**Table 1.** Demographic Information for Participants

Participant pseudonym	Age	Relationship to person with memory loss	Format of interview
Ami	Early 50s	Daughter	Face to face
Cherry	Late 50s	Daughter	Face to face
Colette	Late 50s	Daughter	Online
Dennis	Late 50s	Son	Online
Linus	Late 50s	Son	Online
Lloyd	Early 70s	Son	Face to face
Marcus	Early 40s	Son	Face to face
Mila	Late 30s	Daughter	Online
Sherona	Late 30s	Daughter	Face to face
Shervon	Early 50s	Daughter	Face to face
Winston	Early 50s	Son	Online
Grace, Shirley & Olive	50s & early 70s	Daughter, daughter & Spouse	Face to face

### Interviews

Participants were given the choice of where the interview took place, with the condition that confidentiality could be assured. Five chose to have an online interview, and the others were face-to-face and conducted in a variety of settings, including the participant’s home, community centres, and university premises.

The interview aimed to explore barriers to help-seeking for the memory loss and was shaped by previous research about this issue (Johl et al., 2016; Mukadam et al., 2011a, 2011b, 2015). The interviews explored understanding and beliefs about dementia; cultural expectations, values, and beliefs; and previous experiences of, and attitudes towards, health and social care services. Participants were also asked to reflect on how the experiences of the Windrush Generation may impact help-seeking behaviour. These issues were explored from the perspective of the participant, but also in terms of their understanding of the perspective of the person with the memory loss. Broad open-ended questions were used to introduce an issue before being followed up, if required, with more specific questions and prompts. These broad questions addressed how the family member with the memory loss had reacted and dealt with the memory loss; how the participant themselves had responded; whether cultural beliefs and attitudes influenced these reactions; and their perception of barriers to help-seeking.

### Qualitative Methodology

*Reflexive thematic analysis* (Braun & Clarke, 2022) was used to guide data collection and analysis. It provides the opportunity to use previous research findings to inform these processes, which was considered important because of the aim of building on previous research about the reasons for delayed help-seeking in ethnic minorities. The approach also offers flexibility in terms of the epistemological position of the researcher. In this case, the interest was in providing a description of the participants’ understanding and interpretation of barriers to help-seeking rather than offering an interpretative account of underlying barriers that the participants may not themselves have been aware of. Nevertheless, it is recognised that the assumptions and beliefs of researchers influence the processes of collecting and analysing the data and shape the outcome. The researchers therefore took steps to reflect on what they brought to the process.

Recruitment and interviews were conducted by the lead author who is of African Caribbean heritage. Given the mistrust of services and professionals evident in one of the themes, this was likely to have been important in encouraging participants to engage in the research and to give a less guarded account of their experience. The lead author also has personal experience of help-seeking by family members in the context of symptoms of dementia in the family. The other two authors are of White British heritage. Diversity in heritage may have helped in terms of making the account accessible to a broader audience.

Data analysis followed the steps recommended for reflexive thematic analysis (Braun & Clarke, 2022). First, the lead author listened again to the audio-recordings and re-read the transcripts, making brief notes about potential themes and ideas. Sections of the transcripts that appeared relevant to the research question were highlighted, and initial codes given to these sections. These codes were then grouped together to generate the initial themes. These were then checked against the full transcripts to assess how well they captured the accounts. Themes and the supporting interview extracts were also shared with the other two authors to evaluate the coherence of the themes and how well they were supported by the data. Themes were elaborated and refined in response to these checking processes.

Several steps were taken to enhance the credibility of the data collection and interpretation process. As already described, the authors reflected on how they may have shaped these processes, with the expectation that awareness of this may help prevent excessive distortion in the account; and the coherence and credibility of the themes were evaluated within the whole research team, and elaborations and refinements made in response to this evaluation. The first author also kept a reflexive diary, recording initial reactions to the interviews. This enhanced awareness of what the first author brought to the process of data collection and analysis. This paper makes liberal use of extracts from the interviews to enable the reader to judge how well the themes are based in the participants’ accounts. A participant check was also conducted (McKim, 2023). A summary of the themes was sent out to the participants, and they were asked for feedback about how well the summary captured their experience. Eight participants responded. All gave positive feedback, and no additions or revisions were suggested.

## Results

An overview and brief description of the themes can be found in Table 2. Five of the themes are relatively specific to the culture and ethnicity of the African Caribbean community. More general concerns that would apply across all communities have been grouped into a theme labelled *barriers unrelated to culture or ethnicity*.

**Table 2.** Summary of Themes

**The Windrush Generation: Keep quiet and carry on**
The Windrush Generation have a stoical attitude towards adversity - not making a fuss, relying on one’s own resources, not expecting outside help, carrying on with life. This attitude is born from deprivation and hardship. They had to be self-reliant in the Caribbean. When they arrived in the UK, they faced racism and social exclusion. In the response, they learned to keep a low profile and not to seek help from a society that rejected them.
**Cultural and familial understanding of dementia**
The concept of dementia is not well rooted in Caribbean culture. Forgetfulness likely to be attributed to old age or a harmless individual characteristic.
**Stigma and shame**
If forgetfulness is too severe to be dismissed as old age or a harmless characteristic, then it is a sign of ‘madness’, which is catastrophic and shameful. So, seeking help is delayed and difficulties masked. Shame arises, in part, from the belief that madness is a punishment for bad things done earlier in life.
**Keeping it in the family**
There are cultural and family expectations that care should be provided at home and by the family, specifically the eldest daughter.
**Mistrust of services**
There are concerns that the family member will be mistreated or receive a service that is second-rate or culturally insensitive. So, care needs to be kept within the family to protect the family member. Mistrust arises from personal experience of racism within services and the general historical mistreatment of Black communities.
**Barriers unrelated to culture or ethnicity**
There is a perception that seeking help is not useful because services have little to offer, and there are fears about the financial consequences of receiving a diagnosis of dementia.

### The Windrush Generation: Keep Quiet and Carry On

Participants described a stoical attitude in the African Caribbean community towards adversity in general, labelled by Colette as a ‘keep quiet and carry on’ mentality. Sherona elaborated on this:

There’s been a lot of trauma and I would say there’s a community-based belief that we get on with it, or we cope as much as we can, and that falls to my mom’s generation. We go by the old school we don’t need to talk about these things so we shut up shop and cope with things the best we can. (Sherona)

This suggested that the person with memory loss, and other members of the family, did not make a fuss about the memory loss and tried to carry on as normal, thereby delaying help-seeking.

Participants described how this attitude was born from the deprivation and hardships that the Windrush Generation had to face, both in their country of origin and in the UK. There was little state help in their countries of origin and so families and communities had to rely on their own resources to deal with adversity. This gave them a belief in their own ability to manage difficulties.

She’s Jamaican born. You know, they’ve had their challenges; they’ve come to this country and had more challenges - so that fighting strong willed thing is there, you know. (Colette)

When they migrated to the UK, the Windrush Generation faced racism and exclusion. Winston gave a vivid account of the “everyday occurrences of racism, prejudice and abuse” faced by his parents. They were shunned by the White majority and felt like “outsiders”. To avoid provoking the White majority, they kept a low profile and kept to their own community. State and community facilities and resources were seen as being for the White majority and not for the African Caribbean community. They had to rely on their own resources and did not look for help from a society that rejected them.

My mom’s community, they have lived a life on the periphery and I think that has shaped and forged their decisions in that they think ‘it’s not for me’ or ‘I am not included’ and I think it’s from a culture of when mainstream things were not for them, or [they were] actively being curtailed in their aspirations in needing or wanting to be in places but those places not being receptive. (Sherona)

### Cultural and Familial Understanding of Dementia

Some participants described a lack of knowledge about dementia, and how the concept of dementia itself is not well rooted in Caribbean culture and therefore is less likely to be used to explain memory loss. Instead, forgetfulness is attributed to old age or a harmless trait of the individual, and so help-seeking is again likely to be delayed.

Where he’s from in the Caribbean, it’s not something you would see or label - so culturally it doesn’t fit [Mila]

He [her father] has said a few times you know, when you’re old, you forget stuff. So, yeh, I think he puts it down to age. [Ami]

I first noticed repetitiveness in conversations. She might ask me a question but then within five minutes she would ask me the same question that I’ve already answered. It was simple things like that I first noticed. That was mom’s trait anyway - so you’d almost think, oh it’s just mom being mom. [Dennis]

### Stigma and Shame

When the forgetfulness became so marked that it was difficult to attribute it to harmless causes, participants described how then, in cultural terms, it would be viewed as a sign of being ‘mad’. Madness, in turn, is viewed as catastrophic and shameful. Suggestions that memory loss may be something more than old age or a harmless trait are therefore difficult to accept and more likely to be rejected. Again, this leads to delay in help-seeking.

It’s just the culture in general, they don’t handle mental health or anything like that very well, you know it never has…From a Jamaican perspective, they don’t deal with any type of mental health or accept it. [Ami]

In Jamaica, a couple of doors up from my aunt’s, there’s a family who has a child with severe difficulties and they’ve made a brick house outside the house and the child is locked up in that…I’m just giving you an example of how it’s managed over there. [Ami]

[Her father is a] Typical hard Jamaican man [he says] “nothin’ wrong with mi head - you think I’m going mad”. [Ami]

Memory problems either means related to age but if you start presenting with other types of symptoms like Alzheimer’s, then it’s more like you’re mad. [Mila]

Culturally, for my dad and my Dad’s generation, I think it’s [dementia] something that is seen as ‘life is over for you’. It’s not just a memory problem it’s seen as […] sorry, I have no other way but to say, like you’re mad. [Mila]

Another cultural aspect that was seen to contribute to shame and stigma is the belief that dementia and other forms of ‘madness’ are punishment for bad things the person did earlier in their life. This influenced help-seeking because people are reluctant to talk about the possibility of dementia for fear of this being perceived as an admittance of earlier wrongdoing.

With a lot of African people, you did such and such a thing and now you’re being punished and that’s it. I think that’s one of the reasons why our people are very reluctant to talk about the illness to admit they’ve got illness because it admits that you have done something wrong. It’s like a taboo - you’ve done something wrong. [Lloyd]

As well as delaying initiation of the help-seeking process, the shame associated with dementia could also lead the person with memory loss to try to mask their difficulties even when help is sought. This could lead to a delay in diagnosis and obtaining help.

My dad would arrive at appointments suited and booted, he had his best clothes on, he’s got his best voice to articulate whatever it is he feels there is to articulate and presenting himself well. You know you can’t really hide memory problems but he’s concentrating and using all of his efforts to come across as a well put-together African man in this professional setting. So, it’s hard because my mom is trying to explain the difficulties but, if the person presents in a certain way which is masking all the difficulties, then you’re not getting the help you need. [Mila]

### Keeping it in the Family

Participants described cultural and familial expectations that care should be provided by the family, and particularly by the daughters. This made it less likely that external help and support would be requested because it is seen as less acceptable.

I honestly believe that there’s so much conditioning that goes into the first girl child and it’s almost like this pivotal moment, everything that’s got you to this point has led you to be caregiver number one…I have this conversation with my siblings and they don’t feel they have the same need of duty, I feel duty-bound and that’s a feeling for some reason they don’t have. [Sherona]

I think because of his age as well, he [her father] can be quite sexist and I think, from my point of view, if me and my sister were boys it would be different. I think because we’re girls, it’s an expectation that ‘you’re a girl so you should provide care’. [Cherry]

That’s how my brother sees me: I’m the carer; ‘that’s your job, that’s your duty’. (Shervon).

The converse of the expectation that care should be provided by the family was the expectation that African Caribbean people should not be in residential care.

I don’t know, but I’m just speaking from my own experiences I can’t speak about anyone else’s, but African people don’t tend to go into homes as much as other cultures and culturally I don’t know many people in my culture that have got their own family in a home. Within my culture, they look after them at home and try to protect them that way and I don’t really know anyone that’s in a home. (Shervon).

Do I want to put them in a care home further down the line? […] I don’t really want to, I suppose there is that perception that maybe as African people we don’t put our people in care homes whereas White people might find it a bit easier, more acceptable maybe. [Cherry]

### Mistrust of Services

Participants described a lack of trust in health and social care services, and the fear that the family member would be mistreated or receive a second-rate service because they are Black.

I think cos we’re African, they are just not bothering as much and that’s an attitude that’s just there. Whether that’s an attitude that’s conscious or unconscious, I don’t know but it’s there because we are African. [Shervon]

You know when they do that thing, when you’re asked to complete forms and they ask you what ethnicity you are? I have a feeling that behind the scenes that possibly that is what happens [they realise you are African] - which is why you know, you're left to it, they’re not gonna help. [Colette]

I feel as though some professionals when they are presented with her [participant’s mother] in front of them, unfortunately there’s a prejudice or a bias that comes forth. [They think] “OK, so we can kind of slack we can push back, there’s no urgency that’s needed, there’s no need for us to be on top form”. [Sherona]

These concerns about racial discrimination resulted in a reluctance to engage with services and a belief that care needs to be provided at home as a way of protecting their loved one.

A lot of people in my culture if there’s a problem or a difficulty, they would hide their parents to protect them, they would look after them inside, they wouldn’t tell them [authorities]. They would protect them, they wouldn’t say ‘I’m gonna phone this one, this one, or this one’. You involve too many authorities, then this might happen. So, they try to shield them or protect them. [Shervon]

The mistrust of services arose from personal experiences of racism within services, and the general historical mistreatment of Black communities. An example of the first was provided by Colette, who wondered whether a care company would have handled a potentially serious incident differently had she been White:

I went to my mom’s house and there was a burn mark on the floor. No one had called me to say there was a mark, or if my mom had had an accident. I called the office to ask if both carers had gone in; they said yes and reported no concerns. That was a red flag and really upset me because mom could have been hurt…It makes me wonder if they would have treated me differently if I were White. [Colette]

Some participants discussed the mistrust of services within the context of a more general mistrust of medicine, authority, and government institutions based on historic mistreatment of Black communities. Linus, for example, reflected on how the African Caribbean community feel that society and the medical industry “does not do things in [the African Caribbean community’s] best interests” and that “When you really start to look into the way how society’s been constructed, you start to go, so who is this set up for? It’s not really set up for us”. Some participants provided historical examples of mistreatment:

I would say it’s a difficult one with the community. 100% there’s definitely mistrust and especially in the age of information we know all the Tuskegee experiment, we know that various sort of clinical projects are taking place globally that have had detrimental impacts on people of colour. [Sherona]

Some participants described anxieties about the quality of care their family member would receive in residential care, and a reluctance to use these services because of this. They were concerned about potential racist abuse and a lack of cultural sensitivity.

We did look into African Caribbean care homes, but my partner’s friend said her mom was in one and they were really rude to each other…and then, on the flip side, we were thinking but if mom goes to a predominantly white one, will she be racially abused? [Dennis]

They just generally are not understanding. For example, my dad saying ‘my foot’s hotting me’, to another [White] professional, they wouldn’t understand what he’s on about or where the pain was. You have to understand how they are explaining things, do you know what I mean? Unless there’s someone from an African Caribbean background working there, then they wouldn’t have a clue. [Ami]

Mom was in a home, so my sister wanted us to have a rota so each of us could bring my mom Caribbean food even though we’re paying the home to feed her. [Linus]

### Barriers Unrelated to Culture or Ethnicity

In considering barriers to help-seeking, participants also mentioned issues unrelated to ethnicity and culture. Some suggested that there was little incentive to seek help, particularly in the early stages, because the services had little to offer and functioned poorly.

When the doctor says this is [dementia] and you don’t know where to turn for help, they don’t offer no other help, they don’t say where to go. We’re just at a loose end really. [Grace]

The doctor referred us to [service]. They said they would get in touch with us, but they haven’t. [Olive]

Another issue was concern about the financial implications of there being a diagnosis of dementia, which made some reluctant to involve services.

I think I don’t wanna get people involved because I don’t know what the financial implications are. My dad’s got some finances and I don’t want the government or the housing to take it. So I think I’m just a bit more resistant to give out more information and I don’t know what the repercussions are. Because further down the line, I don’t know, are they gonna come back and say, well, we need to have that off you? [Cherry]

## Discussion

The aim of this research was to gain a better understanding of why people from the African Caribbean community delay seeking help from health and social care services. Delays to the recognition that there was a problem stemmed from the concept of dementia not being well rooted in Caribbean culture, and forgetfulness being attributed, instead, to old age or a harmless trait of the family member. When it was difficult to deny the existence of a problem, there was still a reluctance to acknowledge the possibility of dementia because this is viewed as a form of ‘madness’, and considered shameful and catastrophic. This leads to delays seeking a diagnosis and attempts on the part of the family member to mask their difficulties. Another issue contributing to the reluctance to seek help in the first place is the stoical attitude of the Windrush Generation, that one should not make a fuss about adversity but should carry on with life as normal, and the attitude that they must rely on their own resources to deal with adversity rather than on a society that rejected them.

Once the problem was acknowledged, there is a reluctance to make use of available services. Cultural expectations are that the family member should be cared for at home, and that the oldest daughter should provide this care. There is a concern that the family member will be mistreated or receive a service that is second-rate or culturally insensitive, and therefore care needs to be kept within the family to protect the family member. Participants also highlighted barriers to seeking help that were not related to their culture or ethnicity, specifically the perception that there was little point in the early stages because services had little to offer, and concerns about the financial consequences of a dementia diagnosis.

Several of these findings echo the outcomes from earlier studies about delayed help-seeking amongst ethnic minority communities in the UK (Baghirathan et al., 2020; Berwald et al., 2016; Johl et al., 2016; Mukadam et al., 2011a, 2011b, 2015; Victor et al., 2024). The theme concerning the ‘keep quiet and carry on' attitude of the Windrush Generation was less evident in these earlier studies. This suggests the value of focusing on specific cultural groups, rather than grouping ethnic minorities together; and the value of exploring the impact of migration experiences on help-seeking (Jutlla, 2015; Victor et al., 2024).

The mistrust of services described by the participants, and their concerns about family members receiving inferior treatment because of their ethnicity, reflect a wider problem of perceived racism within health and social care services. For example, in relation to mental health services, people from the African Caribbean community in the UK are more reluctant to engage because of similar concerns about receiving poorer treatment because of their ethnicity (Degnan et al., 2022; Mclean et al., 2003). More broadly, in a 2022 survey, 65% of Black people in Britain reported being discriminated against by healthcare professionals because of their ethnicity (Black Equity Organisation, 2022). This is an international issue, with similar experiences being reported by ethnic minorities across numerous countries and many different health and social care settings (Sim et al., 2021). There is also evidence that this is not simply a perception of discrimination, but that discrimination does occur and leads to significantly poorer health outcomes for those from ethnic minorities (Chauhan et al., 2020; Sim et al., 2021).

Participants reported that, for the Windrush Generation, the experience of racism and general social exclusion led them to rely on their own resources and not to expect help from a society that rejected them. As described in the Introduction, this community was subjected to more recent rejection when the UK government introduced a ‘hostile environment’ policy in 2012 that required people to provide proof of their right to reside in the UK. Many in the Windrush Generation were unable to provide the necessary evidence, in part because the UK government had destroyed the original documentation, and subsequently suffered wrongful deportation and restricted access to services (UK Government, 2024). Against this background, it is not surprising if those belonging to the Windrush Generation and their descendants continue to be mistrustful of services and prefer to rely on their own resources to deal with problems, rather than seeking help from a ‘hostile’ society.

Based on the findings from this and previous studies (e.g., Baghirathan et al., 2020; Berwald et al., 2016; Victor et al., 2024), several suggestions can be made about ways to encourage people of African Caribbean heritage to make more timely use of dementia services. The themes about the cultural and familial understanding of dementia and about the stigma and shame surrounding dementia, indicate the need for raising awareness and understanding of dementia in this community. Such an initiative would need to address directly misconceptions that dementia is a disease of White people (Berwald et al., 2016) and that it is punishment for past sins, and would need to tackle the stigma associated with mental difficulties. Keeping care within the family is not necessarily something to be discouraged, but there are issues to be tackled if this is motivated by a sense of shame and cultural pressure, or by concerns about mistreatment and a lack of cultural sensitivity in available services. Organisations also need to tackle the issue of discrimination and cultural insensitivity within the services they offer.

Plans to tackle these issues need to take account of the mistrust of services and government institutions. In the context of this mistrust, efforts to raise awareness and understanding of dementia, to tackle stigma and shame, and to improve services, are less likely to be effective if they are led by the very institutions that the community mistrusts. Previous research has suggested that, when faced with the challenges of dementia, people from ethnic groups are more likely to turn to voluntary organisations led by members of their own community (Baghirathan et al., 2020). Funding such organisations to take the lead in tackling the issues may be more effective than government-led initiatives. For example, messages about dementia are more likely to be accepted if they come from within the African Caribbean community, rather than mistrusted government organisations; and those from the African Caribbean community are better placed to design dementia services that are acceptable and welcoming to their community.

## Data Availability

Interview transcripts have not been made publicly available because the participants were not asked to provide consent to this.
